# Pressure based MRI-compatible muscle fascicle length and joint angle estimation

**DOI:** 10.1186/s12984-020-00745-8

**Published:** 2020-08-26

**Authors:** Hyungeun Song, Erica Israel, Shriya Srinivasan, Hugh Herr

**Affiliations:** 1 Center for Extreme Bionics, Massachusetts Institute of Technology (MIT) Media Lab, Cambridge, 02139 MA USA; 2 Harvard-MIT Division of Health Sciences and Technology, MIT, Cambridge, 02139 MA USA; 3 Department of Media Arts and Sciences, MIT, Cambridge, 02139 MA USA

**Keywords:** Wearable sensor, Muscle state sensor, MRI-compatible

## Abstract

**Background:**

Functional magnetic resonance imaging (fMRI) provides critical information about the neurophysiology of the central nervous systems (CNS), posing clinical significance for the understanding of neuropathologies and advancement of rehabilitation. Typical fMRI study designs include subjects performing designed motor tasks within specific time frames, in which fMRI data are then analyzed by assuming that observed functional brain activations correspond to the designed tasks. Therefore, developing MRI-compatible sensors that enable real-time monitoring of subjects’ task performances would allow for highly accurate fMRI studies. While several MRI-compatible sensors have been developed, none have demonstrated the ability to measure individual muscle fascicle length during fMRI, which could help uncover the complexities of the peripheral and central nervous systems. Furthermore, previous MRI-compatible sensors have been focused on biologically intact populations, limiting accessibility to populations such as those who have undergone amputation.

**Methods:**

We propose a lightweight, low-cost, skin impedance-insensitive pressure-based muscular motion sensor (pMMS) that provides reliable estimates of muscle fascicle length and joint angle. The muscular motions are captured through measured pressure changes in an air pocket wrapped around the muscle of interest, corresponding to its muscular motion. The muscle fascicle length and joint angle are then estimated from the measured pressure changes based on the proposed muscle-skin-sensor interaction dynamics. Furthermore, we explore an integration method of multiple pMMS systems to expand the sensor capacity of estimating muscle fascicle length and joint angle. Ultrasound imaging paired with joint encoder measurements are utilized to assess pMMS estimation accuracy of muscle fascicle length in the tibialis anterior (TA) and ankle joint angle, respectively, of five biologically intact subjects.

**Results:**

We found that a single pMMS sufficiently provides robust and accurate estimations of TA muscle fascicle length and ankle joint angle during dorsiflexion at various speeds and amplitudes. Further, differential pressure readings from two pMMSs, in which each pMMS were proximally and distally placed, were able to mitigate errors due to perturbations, expanding pMMS capacity for muscle fascicle length and ankle joint angle estimation during the full range of plantar flexion and dorsiflexion.

**Conclusions:**

Our results from this study demonstrate the feasibility of the pMMS system to further be incorporated in fMRI settings for real-time monitoring of subjects’ task performances, allowing sophisticated fMRI study designs.

## Background

Functional magnetic resonance imaging (fMRI) is utilized for investigating brain activity in subjects performing given tasks within specific time frames during a functional scan [1]. fMRI allows for a consecutive time-series of brain activity to be compared with specific tasks and movements, which is critical data for uncovering the connections between motor dysfunction and neural activity. To synchronize fMRI frames with motor task data, and to enable real-time monitoring of subjects’ task performances, various MRI-compatible sensors have been proposed to measure grip force [2], joint position [3–5], and net torque [6–9]. Meanwhile, the exchange of efferent motor signals from the brain and afferent sensory signals from the muscular system, through mechanoneural transduction within muscles, is known to play a critical role in fine motor control [10, 11]. Therefore, the development of a MRI-compatible sensor that provides muscle fascicle length would allow for the assessment of afferent signaling within muscles. Furthermore, for populations such as persons who have undergone amputation, assessment of the subjects’ task performances needs to be based on muscle fascicle movements, independent of joint kinematics and kinetics. Thus, the implementation of a MRI-safe sensor for collecting muscle fascicle data in conjunction with brain activity may be immensely important for advancing our understanding of the complex network between the central and peripheral nervous systems, and evaluating the efficacy of rehabilitation strategies to restore neuromuscular control.

Implantable sonomicrometery crystals have been reported to accurately record real time muscle fascicle length changes. Researchers have used sonomicrometery crystals to track muscle fascicle length and dynamics through direct measurement of crystal activity using a sonomicrometry amplifier [12]. Recently, it has been discovered that magnets can be used in a similar manner. Magnetic fields are able to pass through biological tissues which allows for muscle fascicle length changes to be recorded, via distance tracking between implantable magnets, without a powered system [13]. These methods have been proven successful for highly accurate muscular tracking without being affected by outside noise or skin impedance. However, both require invasive surgery and are strictly limited to a majority of the biologically intact population. Also, magnet tracking still needs to be tested for long term viability in human applications as well as MRI-compatibility.

Sonomyography via ultrasound imaging has also been utilized to derive muscle fascicle length and pennation angle measurements. This has been successful in characterizing muscle architecture for applications including gait abnormality detection, quantifying the dynamics of individual muscles, and measuring proprioceptive intent [14, 15]. An advantage of ultrasound imaging is its ability to isolate muscles of interest, down to individual fascicles, resulting in precise, easy-to-visualize measurements. However, this modality is bound to clinical and laboratory settings, and the quality of the imaging is limited to trained technicians. The reliability of ultrasound imaging may also be diminished due to its reliance on individual interpretation of a given image [16].

For the purpose of avoiding the aforementioned, several efforts have focused on cross-sectional expansion of muscle during its movements [17, 18]. These muscle movement-based methodologies place an air pocket, or force sensitive registers (FSRs), on the muscle of interest, and by tracking cross-sectional expansion via air pressure or contact forces, the muscle activity was estimated. Force myography (FMG) has also been explored for detecting volumetric changes of specific muscles inside a compression garment with the intention of estimating muscle activity [19]. These systems have advantages in terms of practicality for being insensitive to skin impedance changes, and do not require invasive attachment to the skin. In the previously mentioned studies, however, the general estimation strategies of variables such as muscle fascicle length and joint angles, that have non-negligible non-linearity between the sensor outputs and target variable, were not explored. In addition, these studies have neglected muscle-skin interactions and sensor dynamics, e.g. air pocket dynamics and non-linearity of FSRs.

**Fig. 1:**
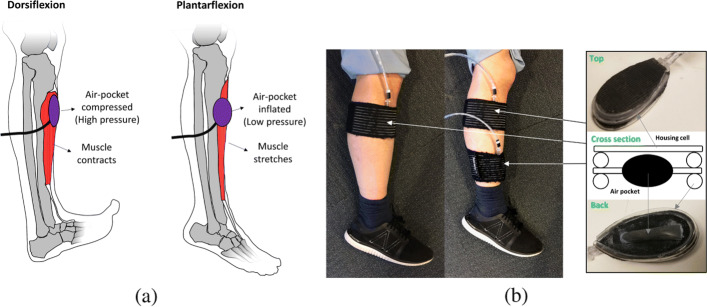
A pressure-based muscular movement sensor (pMMS). The pMMS measures the cross-sectional expansion of the muscle via pressure changes in the air pocket. **a** Mechanism of pMMS, **b** implementation of single-pMMS (left) and differential-pMMS (right)

Although most of the sensors mentioned above have proved successful in clinical settings, they remain futile in conjunction with fMRI for safety and logistical reasons. In this paper, we propose a MRI-safe, non-invasive, cost efficient, pressure-based muscular motion sensor (pMMS) and associated methodology. The pMMS accounts for muscle-skin-sensor dynamics and addresses the ability to estimate changes in muscle fascicle length and joint angles in free-space. Similar to the methodology of the previously mentioned air pocket system, the pMMS utilizes pressure changes in the air pocket due to the cross-sectional expansion of the muscle as shown in Fig. 1a. The proposed method includes a general estimation strategy of target variables that have non-negligible non-linearity with the sensor outputs, to provide robust, real-time estimates of muscle fascicle length and joint angle. These estimations from the pMMS are evaluated by comparative experiments using ultrasound imaging and a joint encoder. Through our investigation, we demonstrate the device’s capability to reject perturbations from surrounding muscles by utilizing the differential pressure of two pMMSs (differential-pMMS), and demonstrate pMMSs’ muscle fascicle length and joint angle estimation accuracy during the full range of motion of the ankle joint. The pMMS may provide several clinically-significant contributions including the ability to synchronize muscle fascicle length and joint angle data with fMRI data in real time while ensuring user comfort and affordability.

## Methods

### Mechanism and design of the pressure-based muscular movement sensor (pMMS)

**Fig. 2:**
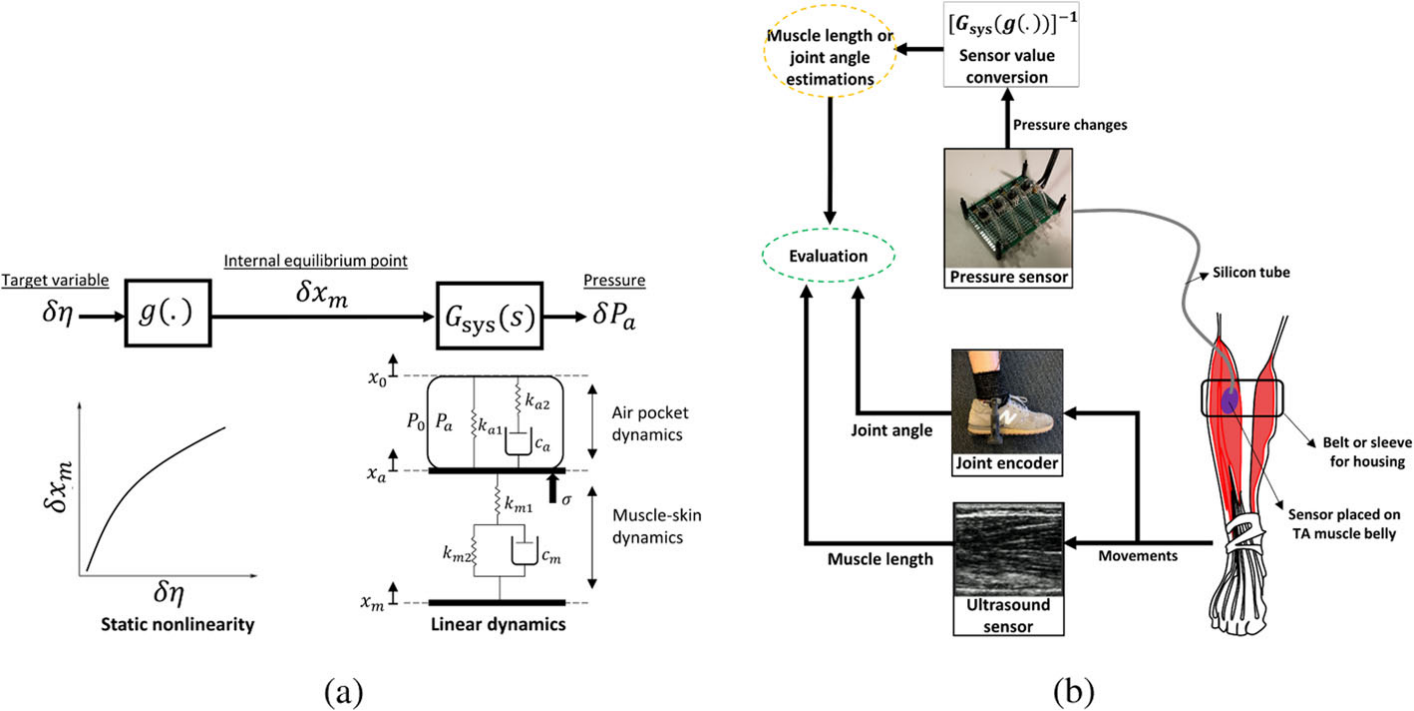
Schematic diagram of pMMS dynamics and evaluation procedures. The pMMS is placed on the tibialis anterior (TA) muscle and secured in place by a bandage or sleeve. The pMMS outputs are converted to the target variable (muscle fascicle length or joint angle) based on the identified muscle-skin-pMMS dynamics consisting of a static non-linearity and a linear system. The performance of pMMS is evaluated by the joint encoder and ultrasound sensor. **a** pMMS dynamics, **b** block diagram of experimental setup

The pressure-based muscular motion sensor (pMMS) consists of a small air pocket (3 ×0.8×0.8 cm) and a housing cell as shown in Fig. 1b. When a muscle is shortened or lengthened, the cross-sectional area of the muscle varies due to changes in the overlapping area of myosin and actin filaments [20], which are biological structures for muscular force production, and muscle fiber pennation angle [21]. By measuring pressure changes within the pMMS wrapped around a muscle of interest, the pMMS reading correlates to the cross-sectional expansion of the muscle, which allows for estimation of the target variables, muscle fascicle length and joint angle, based on the sensor dynamics as shown in Fig. 2a. The housing cell incorporates a double layer structure to minimize pressure changes due to external perturbations and isolates the muscle of interest from surrounding muscles as shown in Fig. 1.

### Modeling

The schematic diagram of pMMS dynamics is shown in Fig. 2a. In this work, an internal equilibrium point *x*_m_ is assumed, which has a static non-linear relationship *g*(.) between its variance *δx*_m_ and that of the target variable *δη*. *x*_m_ is utilized to reflect the *η*-dependent steady pressure of the air pocket *P*_a_. Overall, *δη* results *δx*_m_ based on *g*(.), which propagates through the muscle-skin and deforms the air pocket, causing pressure changes inside of the air pocket *δP*_a_. The pMMS dynamics are derived as follows.

From the ideal gas law, *P*_a_ is described as

1 $$\begin{array}{@{}rcl@{}} P_{\mathrm{a}}&=&\frac{nRT}{A(x_{\mathrm{0}}-x_{\mathrm{a}})} \end{array} $$

where *n*, *R*, and *T* indicates the moles of gas in the air pocket, the gas constant, the absolute temperature, respectively, and *A*, *x*_0_, and *x*_a_ are the contact area, positions of top and bottom of the air pocket, respectively. By taking the derivative of Eq.1, we obtain

2a $$\begin{array}{*{20}l} \delta P_{\mathrm{a}}&=-\frac{nRT}{A(x_{\mathrm{0}}-x_{\mathrm{a}})^{2}}\delta (x_{\mathrm{0}}-x_{\mathrm{a}}) \end{array} $$

2b $$\begin{array}{*{20}l} &=-K_{\mathrm{p}}\delta (x_{\mathrm{0}}-x_{\mathrm{a}})  \end{array} $$

where *K*_p_ indicates $\frac {nRT}{A(x_{\mathrm {0}}-x_{\mathrm {a}})^{2}}$. Because *A* and *n* were constant by the design of pMMS and *T* reached equilibrium with body temperature during trials, *K*_p_ was considered as constant for marginal changes of *δ*(*x*_0_−*x*_a_). The viscoelastic properties of the air pocket are known to be well-described by two springs and a damper [22, 23], which can be described using the force balance equation as

3 $$\begin{array}{@{}rcl@{}} \sigma A &=&A(P_{\mathrm{a}}-P_{\mathrm{0}}) - (k_{\mathrm{a1}}+\frac{k_{\mathrm{a2}}c_{\mathrm{a}}s}{k_{\mathrm{a2}}+c_{\mathrm{a}}s})(x_{\mathrm{0}}-x_{\mathrm{a}}) \end{array} $$

where *σ* and *P*_0_ indicate the contact stress between pMMS and skin surface and barometric pressure, respectively. *k*_a1_, *k*_a2_, and *c*_a_ are the elastic and damping components of the pMMS, respectively. From Eq. 2b and the derivative of Eq. 3 with respect to time, the dynamics between *σ* and *P*_a_ can be described as

4a $$\begin{array}{*{20}l} \delta\sigma &=\delta P_{\mathrm{a}} - \frac{1}{A}(k_{\mathrm{a1}}+\frac{k_{\mathrm{a2}}c_{\mathrm{a}}s}{k_{\mathrm{a2}}+c_{\mathrm{a}}s})\delta(x_{\mathrm{0}}-x_{\mathrm{a}}) \end{array} $$

4b $$\begin{array}{*{20}l} &=G_{\mathrm{s}}(s)\delta P_{\mathrm{a}} \end{array} $$

where *G*_s_(*s*) indicates $1 + \frac {1}{AK_{\mathrm {p}}}(k_{\mathrm {a1}}+\frac {k_{\mathrm {a2}}c_{\mathrm {a}}s}{k_{\mathrm {a2}}+c_{\mathrm {a}}s})$. Additionally, muscle-skin dynamics are known to have three distinguishable mechanical behaviors: the pure elastic, viscoelastic, and constant creep phase [24, 25]. However, the constant creep phase only becomes dominant when deformation of the muscle-skin remains constant for long periods, which is difficult to be performed by voluntary muscle contraction. This high order property is further regressed by air pocket dynamics, thereby we assume that the constant creep phase is negligible. Then, the muscle-skin dynamics are described by an elastic component, *k*_m1_, serially connected to another elastic component, *k*_m2_, and a damper, *c*_m_, to address the pure elastic and viscoelastic phases, or

5a $$\begin{array}{*{20}l} \delta \sigma &=\frac{1}{A}\frac{k_{\mathrm{m1}}(k_{\mathrm{m2}}+c_{\mathrm{m}}s)}{(k_{\mathrm{m1}}+k_{\mathrm{m2}}+c_{\mathrm{m}}s)}\delta(x_{\mathrm{a}}-x_{\mathrm{m}}) \end{array} $$

5b $$\begin{array}{*{20}l} &=G_{\mathrm{m}}(s)(\frac{1}{K_{\mathrm{p}}}\delta P_{\mathrm{a}}+\delta (x_{\mathrm{0}}-x_{\mathrm{m}}))  \end{array} $$

where *G*_m_(*s*) indicates $\frac {1}{A}\frac {k_{\mathrm {m1}}(k_{\mathrm {m2}}+c_{\mathrm {m}}s)}{(k_{\mathrm {m1}}+k_{\mathrm {m2}}+c_{\mathrm {m}}s)}$ and *x*_m_ represents the *η*-dependent internal equilibrium point of the muscle-skin dynamics. Eq. 5b was derived by the combination of Eq. 2b and 5a, putting *x*_a_−*x*_m_=−(*x*_0_−*x*_a_)+*x*_0_−*x*_m_. Then, from Eq. 4b and 5b, the overall muscle-skin-pMMS dynamics are described as

6a $$\begin{array}{*{20}l} \delta P_{\mathrm{a}} &= \frac{K_{\mathrm{p}}G_{\mathrm{m}}(s)}{G_{\mathrm{m}}(s) - K_{\mathrm{p}}G_{\mathrm{s}}(s)}\delta (x_{\mathrm{m}}-x_{\mathrm{0}}) \end{array} $$

6b $$\begin{array}{*{20}l} &=G_{\text{sys}}(s)\delta (x_{\mathrm{m}}-x_{\mathrm{0}}). \end{array} $$

Because the air pocket is shielded by a double layer housing cell, the external perturbations to *δP*_a_, e.g. the muscular movements of surrounding muscles, and external forces, are negligible, i.e. *δx*_0_=0. To enhance generality of the proposed model, the *η*- *x*_m_ relationship is not restricted by the linearity assumption; the static non-linearity *g*(.) is assumed between *δx*_m_ and *δη*, or

7 $$\begin{array}{@{}rcl@{}} \delta x_{\mathrm{m}}&= g(\delta\eta).  \end{array} $$

Then, as shown in Fig. 2a, the *η*- *P*_a_ relationship is described as

8 $$\begin{array}{@{}rcl@{}} \delta P_{\mathrm{a}}=G_{\text{sys}}(s)g(\delta\eta).  \end{array} $$

Therefore, *δη* can be estimated from *δP*_a_ as

9 $$\begin{array}{@{}rcl@{}} \delta \eta = g^{-1}(G_{\text{sys}}^{-1}(s)\delta P_{\mathrm{a}}).  \end{array} $$

Note that the inverse model of pMMS dynamics for the variable estimation can be described as the linear system $G_{\text {sys}}^{-1}$ and the static non-linearity property *g*^−1^(.), or the Wiener system.

### System identification

In this work, the system identification of pMMS is conducted by the revision of the Wiener system identification methodology addressed in [26]. Note that, because both *G*_s_ and *G*_m_ are first order dynamics with a single zero (Eq. 6a), $G_{\text {sys}}^{-1}(s)$ is described as a second order system with two zeros, or

10 $$\begin{array}{@{}rcl@{}} G_{\text{sys}}^{-1}(s)=\frac{a_{2}s^{2}+a_{1}s+a_{0}}{s^{2}+b_{1}s+b_{0}} \end{array} $$

where *a*_i_ and *b*_j_ indicate coefficients. Then, the linear dynamics $G_{\text {sys}}^{-1}(s)$ and static non-linearity *g*^−1^(.) of Eq. 9 are identified by the following process:

1. Estimate the best fit for the linear dynamics $\hat {G}_{\text {sys}}^{-1}(s)$ from *δP*_a_ and *δη*.
2. Estimate output of the linear dynamics $\hat {y}$ from the convolution of $\hat {G}_{\text {sys}}^{-1}(s)$ with *δP*_a_.
3. Fit a high-order polynomial $g_{\mathrm {p}}^{-1}(.)$ between $\hat {y}$ and *δη*.
4. Re-estimate $\hat {y}$ from *η* via the inverse of the polynomial
5. Re-estimate $\hat {G}_{\text {sys}}^{-1}(s)$ from *δP*_a_ and $\hat {y}$.
6. Re-estimate $\hat {y}$ from the convolution of $\hat {G}_{\text {sys}}^{-1}(s)$ with *δP*_a_.
7. Go to the step (3) (another iteration) or go to the step (8) (final estimation)
8. Create lookup table $g_{\mathrm {l}}^{-1}(.)$ to model high order nonlinearity between $g_{\mathrm {p}}^{-1}(\hat {y})$ and *δη*.

Note that the identified static non-linearity $\hat {g}^{-1}$ is determined as $g_{\mathrm {l}}^{-1}(g_{\mathrm {p}}^{-1}(.))$. In addition, because *δP*_a_ has strong correlations with the muscle fascicle length *l*_m_ and joint angle *θ*, we assume a second order polynomial for *g*_p_(.) during iterative system identification procedure.

### Model verification of pMMS

The pMMS estimation of muscle fascicle length and joint angle is evaluated by comparing fascicle length and joint angle measurements via the ultrasound sensor and joint encoder as shown in Fig. 2b. The proposed model is then verified by measuring muscle motion of the tibialis anterior (TA). Five subjects (S1-S5) participated in this study as shown in Table 1. All experiments were carried out with informed consent at the Massachusetts Institute of Technology (MIT), under the approval of the Committee on the Use of Humans as Experimental Subjects (COUHES). The pMMS was affixed to the skin overlying the muscle using a compression sleeve or elastic wrap depending on the calf circumference and comfort of each subject. The pMMS placement was optimized to maximize the pressure sensor outputs during dorsiflexion. For system identification and evaluation of the pMMS estimations of the joint angle *θ* and muscle fascicle length *l*_m_, the joint encoder and ultrasound sensor were utilized as shown in Fig. 2b. For all trials, the subjects were asked to wear the joint encoder on their ankle to have the measurements of *θ* correspond to the pMMS outputs. Due to practical constraints surrounding ultrasound sensor placement alongside the pMMS, *δl*_m_ is calculated from *δθ* values based on a lookup table capturing *θ* and *l*_m_ which were identified prior to the trials without the pMMS. In addition, the *δl*_m_ was normalized by the full range of *δl*_m_ to evaluate over different individuals, referred to as *Δε*_m_.

**Table 1:** Subject descriptions

Subject	Gender	Height	Weight
S1	Male	173cm	76kg
S2	Female	165cm	40kg
S3	Female	167cm	48kg
S4	Male	196cm	100kg
S5	Male	188cm	98kg

To validate the proposed pMMS model, patients were asked to dorsiflex their foot, activating only the TA, from neutral position to full dorsiflexion. Full range of motion (ROM) that involves full plantar flexion would have introduced external perturbations from surrounding muscles into the measurement which will be detailed in the later portion of this report. In the first experiment, half-amplitude sweep trials, subjects were asked to dorsiflex at approximately 30, 60, and 90 percent of their full range of dorsiflexion at a steady pace. However, no specific joint trajectory was provided for the subjects to allow variance in the movements between each cycle. This range of movement enabled us to fit system parameters across a larger range of muscle movements. For the second experiment, half-frequency sweep trials, the subjects repeatedly performed dorsiflexion in the following pattern of paces: slow, fast, and hold; in which they stopped at full dorsiflexion for at least 3 seconds. Similar to the half-amplitude sweep trials, no specific trajectory guidelines were given to the subjects. Two data sets of each experimental scheme were collected. The system identification was conducted based on an integrated data set which consists one of two data set of both half-amplitude and half-frequency sweep trials. Model verification was conducted by comparing the estimates of the target variables, based on the identified system model, to their measured values of the trials that were not used in the system identification procedures.

### External perturbations from surrounding muscles

**Fig. 3:**
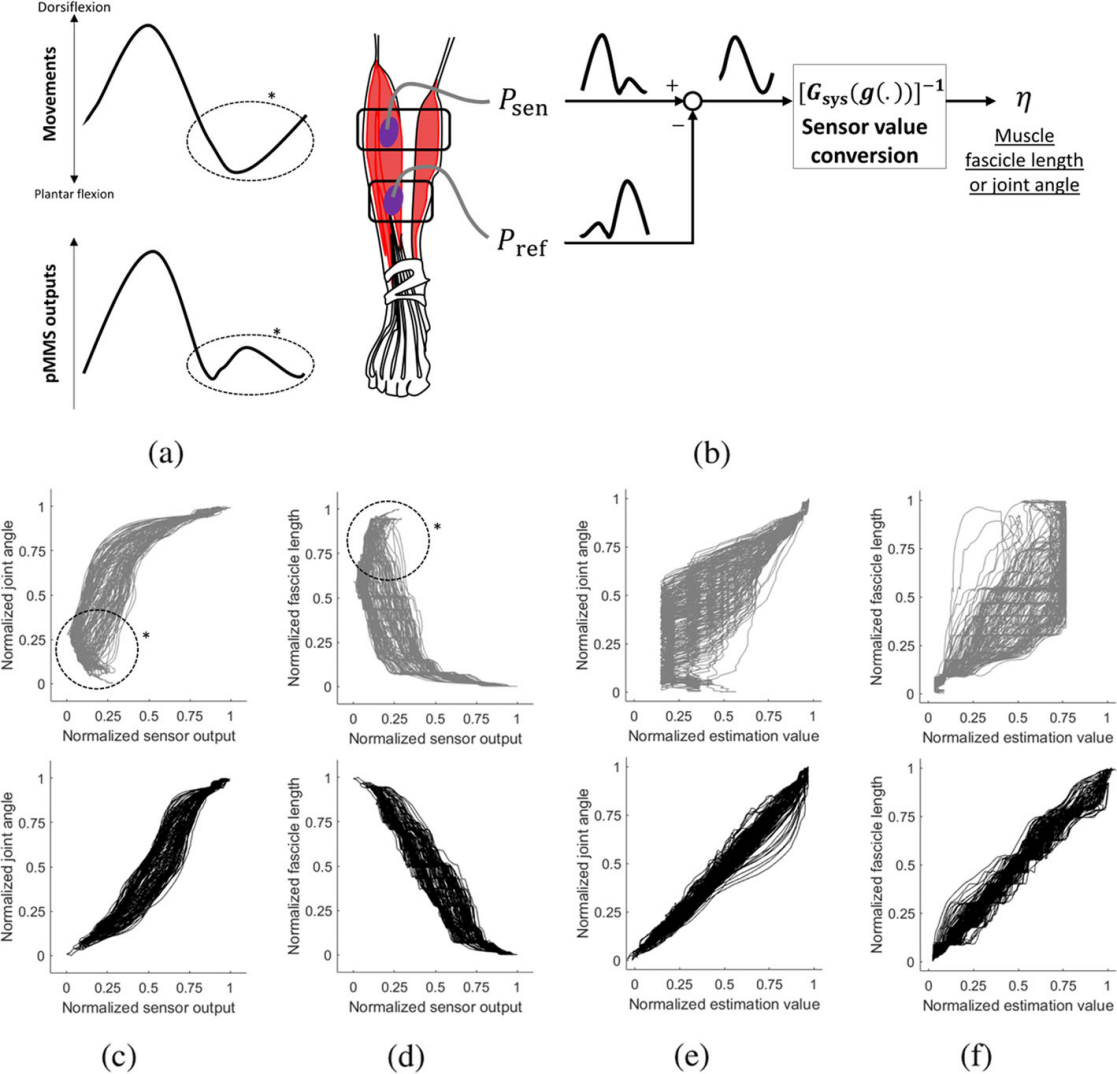
External perturbations to the pMMS at extreme range of motion (ROM). The pMMS outputs do not uniformly correlate with the muscular movements at extreme ROM (circled with *). These perturbations are compensated by a differential-pMMS. **a** Typical single-pMMS outputs from the TA during dorsiflexion and plantar flexion, **b** schematic diagram of the differential-pMMS, **c** representatives of singe-pMMS (top) and differential-pMMS (bottom) outputs to varying joint angles, **d** representatives of singe-pMMS (top) and differential-pMMS (bottom) outputs to varying muscle fascicle length **e** representative linear plot of singe-pMMS (top) and differential-pMMS (bottom) estimation of varying joint angles, **f** representative linear plot of singe-pMMS (top) and differential-pMMS (bottom) estimation of varying muscle fascicle length

In the previous experimental scheme, the pMMS model was evaluated under a limited ROM (half-amplitude and half-frequency sweep trials) to minimize external perturbations and focus on the sensor model verification. However, in practice, the muscular movements of surrounding muscles, e.g. peroneus longus (PL), tibialis posterior (TP), and antagonist muscles, e.g. gastrocnemius (GAS), may not be negligible and cause large estimation errors particularly at the extreme ends of ROM. A typical pMMS readings during full dorsiflexion and plantar flexion is demonstrated in Fig. 3a. During extreme plantar flexion, the pressure values do not uniformly correlate with the muscular movements due to external perturbations, which are marked as asterisks (∗). For these values, because the pMMS cannot recognize whether the current pressure readings correspond to a small dorsiflexion or extreme plantar flexion, the external perturbations could cause significant errors in the both system identification and variable estimation as shown in Fig. 3c-f.

To investigate the effect of these external perturbations, all five subjects (S1–S5) were asked to perform two types of full range dorsiflexion and plantar flexion: full-amplitude sweep and full-frequency sweep trials. During the full-amplitude sweep trials, the subjects repeatedly performed dorsiflexion and plantar flexion, gradually increasing the displacement from neutral position with each cycle. For the full-frequency sweep trials, the subjects were asked to repeat the same task as the half frequency sweep trial but with their full ROM (full dorsiflexion to full plantar flexion). No specific joint trajectory was given for any trials to allow their dorsiflexion and plantar flexion to be varied. At least two trials for each type of task were obtained for system identification and validation. The system identification and data processing were conducted in a similar manner to the previously described method.

### External perturbation compensation by differential-pMMS

To compensate external perturbations from surrounding muscles, we use an additional pMMS as a reference value, and utilize the differential pressure of two pMMSs (differential-pMMS) to amplify the pressure changes by the muscular movement of interest and cancel out external perturbations as shown in Fig. 3b. This provides uniform positive or negative correlation with the target variables as shown in Fig. 3c-f. Thus, for the muscles that are affected by non-negligible external perturbations, the differential pressure *δP*_diff_ can be used as

11 $$\begin{array}{@{}rcl@{}} \delta P_{\text{diff}}&=& \delta P_{\text{sen}}-\delta P_{\text{ref}} \end{array} $$

12 $$\begin{array}{@{}rcl@{}} \delta \eta &=& g^{-1}\left(G_{\text{sys}}^{-1}(s)\delta P_{\text{diff}}\right).  \end{array} $$

to estimate *δη* where *δP*_sen_ and *δP*_ref_ indicate the pressure values of the pMMS on the muscle belly and reference point, respectively.

## Results

### Identified system dynamics

The identified linear system dynamics, $G_{\text {sys}}^{-1}(s)$, are given in Table 2. See Supplemental information for subject-specific dynamics. Large variances were found in the parameters of identified system dynamics between subjects. These variances are due to the sensitivity of the pMMS to sensor placements and tension of the sleeve to hold the sensor, which vary *K*_p_, *k*_m1_, *k*_m2_, *c*_m_ as well as static nonlinearity between the target variable, *δη*, and the measured pressure, *δP*_a_.

**Table 2:** Identified linear system dynamics, $G_{\text {sys}}^{-1}(s)$, from half-amplitude and half-frequency (H.H.) and full-amplitude and full-frequency (F.F.) trials for joint angle (J.A.) and fascicle length (F.L.) estimations

Type	*a*_2_	*a*_1_	*a*_0_	*b*_1_	*b*_0_
H.H.J.A. (single)	165.5 ± 29.8	1823 ± 1587	1836 ± 1807	9.7 ± 8.5	8.5 ± 8.3
F.F.J.A. (single)	140.2 ± 56.6	5115 ± 5049	682.2 ± 578.0	11.6 ± 10.2	8.6 ± 5.6
F.F.J.A. (differential)	143.9 ± 53.5	7189 ± 2359	145.5 ± 87.7	26.1 ± 8.7	1.3 ± 0.8
H.H.F.L. (single)	-47.7 ± 19.6	-202.4 ± 178.6	-183.5 ± 161.4	2.5 ± 2.0	1.8 ± 1.5
F.F.F.L. (single)	-12.0 ± 4.8	-736.1 ± 537.3	-791.7 ± 756.1	14.0 ± 7.9	21.7 ± 12.9
F.F.F.L. (differential)	-12.1 ± 5.5	-1083 ± 536	-8833 ± 5796	27.3 ± 5.1	141.6 ± 105.3

Data are reported as mean ± 1 S.E.M.

### Model verification of pMMS

**Fig. 4:**
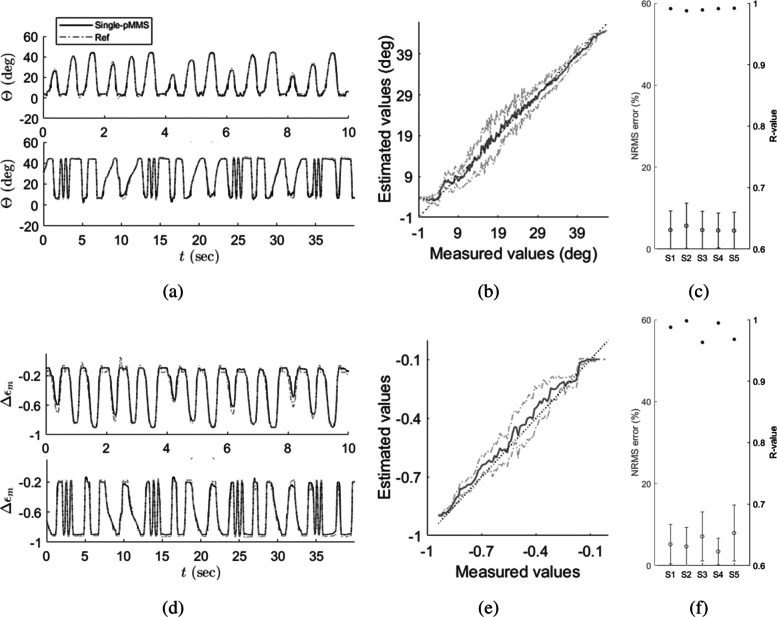
Model validation results. **a** Representative joint angle estimation result from half-amplitude (top) and half-frequency (bottom) sweep trials (S1), **b** representative linearity plot of joint angle estimation (S1), **c** summary of NRMS estimation errors (error bars) and R-values (*) of joint angle estimation (S1-S5), **d** representative muscle fascicle length estimation result from half-amplitude (top) and half-frequency (bottom) sweep trials (S1), **e** representative linearity plot of muscle fascicle length estimation (S1), **f** summary of NRMS estimation errors (error bars) and R-values (*) of muscle fascicle length estimation (S1-S5). The dotted lines and solid lines in the estimation results indicate the measured values and estimation values from pMMS outputs, respectively. For the linearity plots, the solid lines and dotted lines indicate the mean and 1 S.D. of the pMMS linearity, respectively

The model verification results are shown in Fig. 4 and given in Table 3. For the joint angle estimation results, normalized root mean square (NRMS) errors were calculated by normalizing the root mean square (RMS) error in the ROM, which is referred to as *e*_J_. Meanwhile, because *Δε*_m_ is already the normalized variable, the RMS errors of *Δε*_m_ were used for the evaluation, which is referred to as *e*_M_. Additionally, the correlation coefficient (R-values) for the joint angle R_J_ and muscle fascicle length estimations R_M_ are calculated. The *e*_J_ and *e*_M_ are shown as 4.8 ± 0.5% and 5.7 ± 1.8% over the five subjects, respectively. Similarly, the R_J_ and R_M_ indicate strong linearity. The R_J_ and R_M_ are 0.990 ± 0.002 and 0.980 ± 0.014, respectively. Therefore, while the proposed model may simplify complex muscle-skin-pMMS dynamics, the current pMMS model was able to provide muscle fascicle length and joint angle estimation within 5–6% NRMS errors, suggesting validity of the proposed model for the motor tasks at different amplitudes and speeds tested here.

**Table 3:** NRMS errors (*e*_J_, *e*_M_) and R-values (R_J_, R_M_) of single-pMMS half-amplitude and half-frequency sweep trials (S.H.H), single-pMMS full-amplitude and full-frequency sweep trials (S.F.F.), and differential-pMMS full-amplitude and full-frequency sweep trials (D.F.F.)

Type	*e*_J_	*e*_M_	R_J_	R_M_
S.H.H.	4.8 ± 0.5%	5.7 ± 1.8%	0.990 ± 0.002	0.980 ± 0.014
S.F.F.	13.9 ± 1.7%	11.9 ± 2.0%	0.884 ± 0.036	0.923 ± 0.016
D.F.F.	7.0 ± 2.5%	6.4 ± 1.2%	0.971 ± 0.018	0.979 ± 0.010

Data are reported as mean ± 1 S.D.

### External perturbations from surrounding muscles

**Fig. 5:**
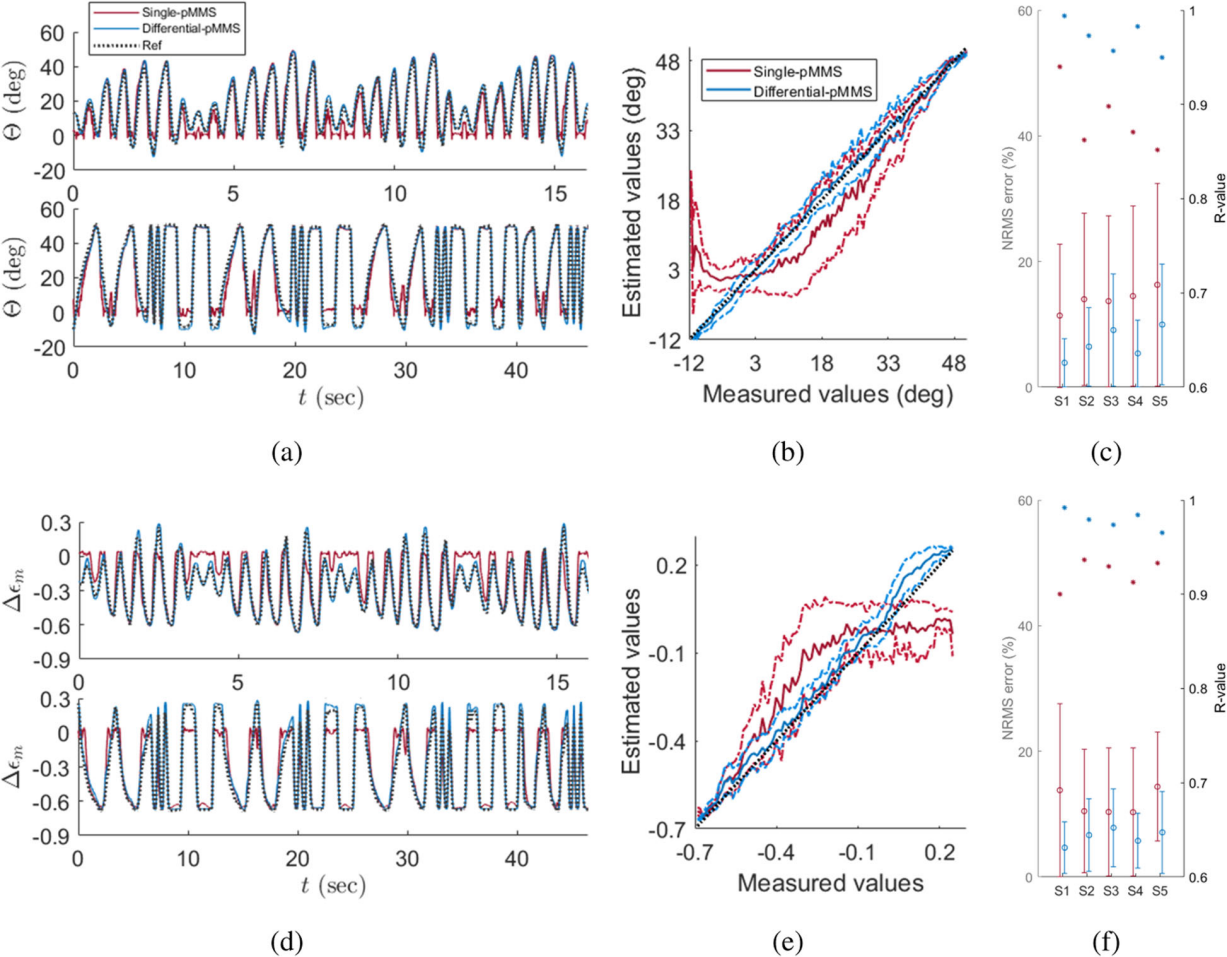
Comparison of single-pMMS and differential-pMMS performances for full-amplitude and full-frequency trials. **a** Representative joint angle estimation result from full-amplitude (top) and full-frequency (bottom) sweep trials (S1), **b** representative linearity plot of joint angle estimation (S1), **c** summary of NRMS estimation errors (error bars) and R-values (*) of joint angle estimation (S1-S5), **d** representative muscle fascicle length estimation result from full-amplitude (top) and full-frequency (bottom) sweep trials (S1), **e** representative linearity plot of muscle fascicle length estimation (S1), **f** summary of NRMS estimation errors (error bars) and R-values (*) of muscle fascicle length estimation. The dotted lines and solid lines in the estimation results indicate the measured values and estimation values from pMMS outputs, respectively. For the linearity plots, the solid lines and dotted lines indicate the mean and 1 S.D. of the pMMS linearity, respectively

The pMMS performance under the external perturbations is shown in Fig. 5 and exhibited in Table 3. Comparing the results of half-amplitude and half-frequency sweep trials, the *e*_J_ and *e*_M_ of the full-amplitude and full-frequency sweep trials drastically increased to 13.9 ± 1.7 *%* and 11.9 ± 2.0%, respectively. Additionally, the R_J_ and R_M_ decreased to 0.884 ± 0.036 and 0.923 ± 0.016, respectively. As described in the “Methods” section, these deteriorations of pMMS performance are due to external perturbations from surrounding muscles as well as insensitivity of pMMS outputs to muscular movements around neutral position as shown in Fig. 3.

### External perturbation compensation by differential-pMMS

**Fig. 6:**
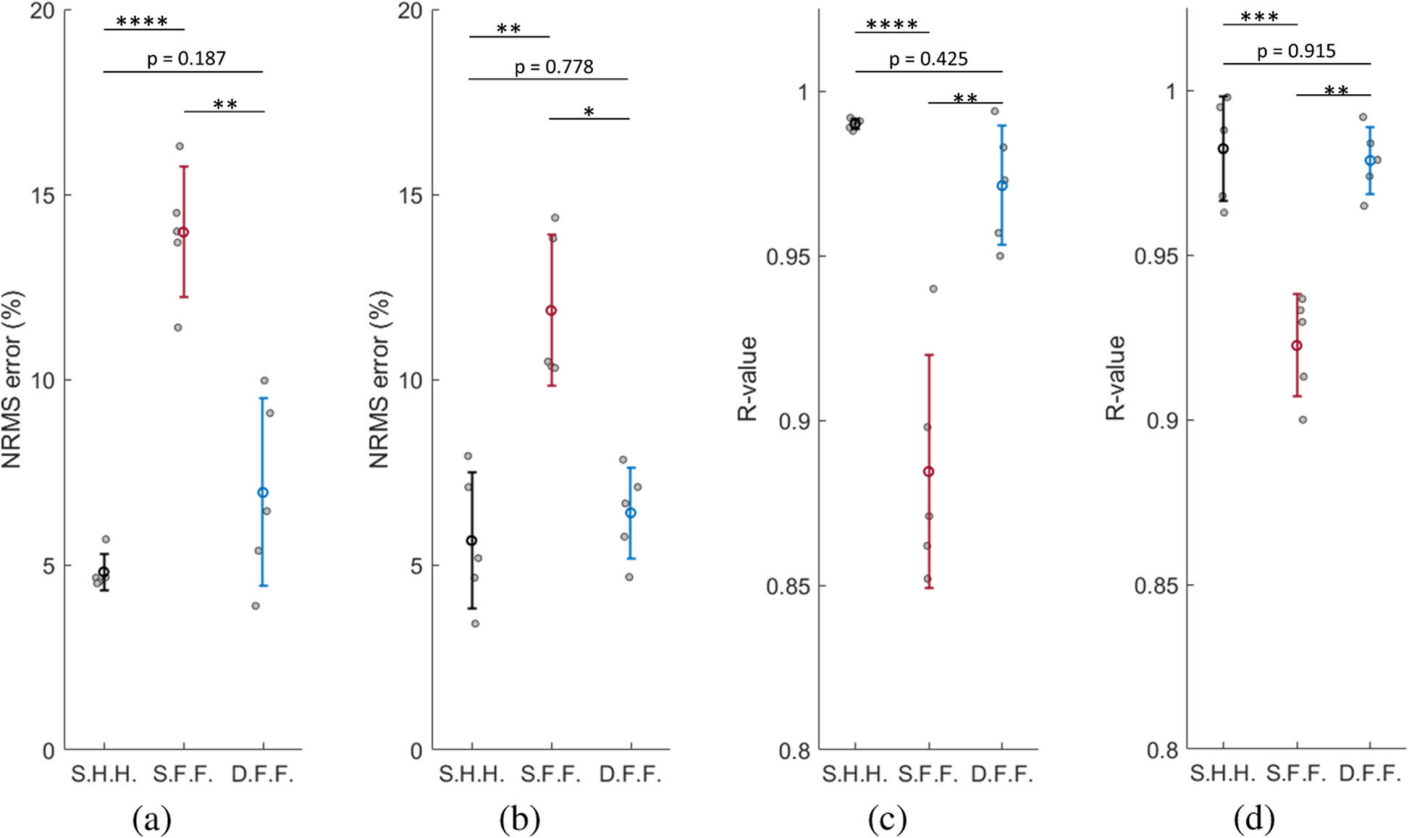
Evaluation of single-pMMS and differential-pMMS configurations. **a** and **b** show NRMS errors of single-pMMS half-amplitude and half-frequency sweep trials (S.H.H), single-pMMS full-amplitude and full-frequency sweep trials (S.F.F.), and differential-pMMS full-amplitude and full-frequency sweep trials (D.F.F.). **c** and **d** indicate R-value of S.H.H., S.F.F., and D.F.F. Data are reported as mean ± 1 S.D. Asterisks above bars indicates significant differences between different pMMS configurations. * *p*<1×10^−3^, ** *p*<5×10^−4^, *** *p*<1×10^−4^, **** *p*<5×10^−5^, one-way ANOVA with Tukey-Kramer multiple comparison tests. Where no significance was seen, a *p* value is shown

The differential-pMMS system was verified by the full-amplitude and frequency sweep trials which demonstrates that the differential-pMMS effectively compensates for the external perturbations (Figs. 3 and 5, Table 3) and improves *e*_J_ and *e*_M_ to 7.0 ± 2.5% and 6.4 ± 1.2%, respectively. Also, the differential-pMMS estimation results show strong linearity to the target variables which R_J_ and R_M_ are 0.971 ± 0.018 and 0.979 ± 0.010, respectively. To further address the affect of differential-pMMS, one-way ANOVA with Tukey-Kramer multiple comparison tests were applied to *e*_J_, *e*_M_, R_J_, and R_M_ of different pMMS configurations which are shown in Fig. 6. No significant differences were found between single-pMMS half-amplitude and half-frequency (S.H.H.) and differential-pMMS full-amplitude and full-frequency (D.F.F.) trials at all matrices including *e*_J_, *e*_M_, R_J_, and R_M_. However, significant differences were found between single-pMMS full-amplitude and full-frequency (S.F.F.) and the other two configurations at all matrices. These results indicate that the differential-pMMS configuration is effective in compensating for external perturbations and successfully provides reliable joint angle and muscle fascicle length estimation during the full ROM at the ankle.

## Discussion

Throughout this work, we have investigated the characteristics and capability of the pMMS to provide muscle fascicle length and joint angle estimation. Using silicon tubing (Fig. 2b), the current pMMS design allows for implementation of the electronics outside of an fMRI scanner room, while keeping the air pocket attached to the patient, demonstrating its viability as a MRI-compatible sensor platform. Moreover, because the pMMS can be easily applied to the muscle of interest without being invasively attached to skin, the pMMS provides superior advantages in terms of practicality. Although high-order muscle-skin interaction dynamics were simplified by neglecting ‘creep’ dynamics, a single pMMS successfully provided comparable estimates to actual measurements of joint angle and muscle fascicle length during active dorsiflexion of all five participants within different speeds and amplitudes as shown in Fig. 4. To overcome large estimation errors resulting from perturbation of surrounding muscles during plantar flexion, shown in Fig. 5, we explored the integration of multiple pMMS to provide estimates of muscle fascicle length and joint angle. By having one pMMS at both the proximal and distal ends of the TA, we found that the estimated joint angle and muscle fascicle length from differential-pMMS readings were comparable to that of the measurements from the joint encoder and ultrasound imaging, expanding pMMS capacity over a full ROM. Therefore, the pMMS may lead to further understanding of CNS and muscle sensory feedback interactions, as well as enhanced rehabilitative strategies for populations with sensory-motor dysfunction. Although the current pMMS poses sufficiency of its use in fMRI settings for real-time monitoring of a subject’s task performance, more general application usage, such as human motor intent estimation for assistive device control and clinical measurements in rehabilitation settings, needs further investigation. Because muscle mechanical properties are also affected by the level of muscle activation, the current pMMS systems may be limited in providing estimates during isometric contraction or passive stretching movements which is commonly performed in rehabilitation and physical therapy settings. To overcome these potential limitations, the incorporation of EMG data in conjunction with pressure data may be used to enhance the usability of the pMMS outside of fMRI applications. In this scenario, EMG data can be utilized to decouple muscle fascicle length and muscle activity from pressure measures, applying advanced sensor fusion methodologies, e.g. Kalman filter [27], fuzzy logic [28].

## Conclusion

In this work, the pMMS design and associated methodology are proposed. Through several diverse trials, investigating the pMMS’s capability to estimate muscle fascicle dynamics under various movement frequencies and joint angle amplitudes, we report high prediction accuracy of joint position and muscle fascicle length. The pMMS poses as a reliable, MRI-safe, wearable sensor for measuring muscle fascicle length and dynamics in populations including persons with amputation or motor dysfunction because of its ability to perform independently from joint angle measurements. The application of the pMMS system in conjunction with fMRI data of brain activity may lead to novel discoveries for the augmentation of rehabilitation and mobility. Future work will include the investigation of pMMS usage in non-MRI settings and the incorporation of EMG for more accurate muscle dynamic measures. Additionally, the pMMS will be tested during rehabilitation appropriate tasks such as passive stretching and weight-bearing activity.

## Additional material

Additional file 1 **Supplemental information 1** Identified subject-specific pMMS dynamics. The parameters of identified linear system dynamics and static non-linearity are reported. **Supplemental information 2** Joint angle and muscle fascicle length estimation results of all five participants. The estimation results of joint angle and muscle fascicle length of all five participants are included. NRMS error and R-value of joint angle and muscle fascicle length estimation results are reported.

## Data Availability

All data are available in the main text or the supplemental information. Any code used for the processing of data in this manuscript can be obtained from the authors upon reasonable request.

## Abbreviations

fMRI: Functional Magnetic Resonance Imaging
MRI: Magnetic Resonance Imaging
pMMS: Pressure-based Muscular Motion Sensor
TA: Tibialis Anterior
sEMG: Surface Electromyography
FMG: Forcemyography
FSR: Force Sensitive Registers
MIT: Massachusetts Institute of Technology
COUHES: Committee on the Use of Humans as Experimental Subjects
ROM: Range Of Motion
PL: Peroneus Longus
TP: Tibialis Posterior
GAS: Gastrocnemius
NRMS: Normalized Root Mean Square
RMS: Root Mean Square
