# Supplementary material for: Pressure based MRI-compatible muscle fascicle length and joint angle estimation
Source: J Neuroeng Rehabil. 2020 Aug 26;17:118. doi: 10.1186/s12984-020-00745-8 (PMC7449022; doi:10.1186/s12984-020-00745-8)
Supplement: Supplementary file 1 — Supplemental information 1 Identified subject-specific pMMS dynamics. The parameters of identified linear system dynamics and static non-linearity are reported. Supplemental information 2 Joint angle and muscle fascicle length estimation results of all five participants. The estimation results of joint angle and muscle fascicle length of all five participants are included. NRMS error and R-value of joint angle and muscle fascicle length estimation results are reported. [file 12984_2020_745_MOESM1_ESM.docx]

**Supplemental Information**

1. **Identified subject-specific pMMS dynamics.**
2. **Subject-specific linear dynamics from half-amplitude & half-frequency (H.H.) and full-amplitude & full-frequency (F.F.) trials for joint angle (J.A.) and fascicle length (F.L.) estimations.**

|  | Subjects | $a_{2}$ | $a_{1}$ | $a_{0}$ | $b_{1}$ | $b_{0}$ |
| --- | --- | --- | --- | --- | --- | --- |
| H.H.J.A. (single) | S1 | 169.3 | 95.8 | 84.0 | 0.7 | 0.6 |
|  | S2 | 222.3 | 135.1 | 18.8 | 0.5 | 0.1 |
|  | S3 | 212.7 | 12.1 | 0.8 | 0 | 0 |
|  | S4 | 168.4 | 720.2 | 13.8 | 3.7 | 0.1 |
|  | S5 | 54.7 | 8150 | 9064 | 43.6 | 41.5 |
| F.F.J.A.  (single) | S1 | 229.4 | 0 | 60.2 | 0 | 0.3 |
|  | S2 | 250.4 | 5.5 | 17.5 | 0 | 0.1 |
|  | S3 | 4.1 | 41.1 | 67.4 | 5.5 | 11.8 |
|  | S4 | 0 | 25310 | 2987 | 52.2 | 29.1 |
|  | S5 | 217 | 219.3 | 278.8 | 0.5 | 1.6 |
| F.F.J.A.  (differential) | S1 | 47.8 | 8415 | 0 | 39.3 | 0 |
|  | S2 | 172.8 | 3677 | 372.7 | 10.2 | 2.4 |
|  | S3 | 329.8 | 31.2 | 7.4 | 0 | 0 |
|  | S4 | 32.1 | 12810 | 347.4 | 41.1 | 4.0 |
|  | S5 | 137.2 | 11010 | 0 | 39.9 | 0 |
| H.H.F.L.  (single) | S1 | -14.3 | -5.5 | -4.8 | 0.5 | 0.4 |
|  | S2 | -111.5 | -1.2 | -84.6 | 0 | 0.7 |
|  | S3 | -0.7 | -0.1 | 0 | 0 | 0 |
|  | S4 | -48.0 | -91.9 | -2.1 | 1.7 | 0.1 |
|  | S5 | -63.9 | -913.5 | -826 | 10.3 | 8.0 |
| F.F.F.L.  (single) | S1 | -2.5 | -5.5 | -26.8 | 1.6 | 9.4 |
|  | S2 | -25.7 | -880.8 | -3815 | 15.3 | 72.3 |
|  | S3 | -0.1 | -1.9 | -1.7 | 8.0 | 10.2 |
|  | S4 | -14.6 | -2776 | -111.8 | 43.9 | 16.3 |
|  | S5 | -17.1 | -16.4 | -3.3 | 1.1 | 0.3 |
| F.F.F.L.  (differential) | S1 | -27.6 | -782.4 | -28670 | 24.3 | 543.0 |
|  | S2 | 0 | -3093 | -15490 | 40.8 | 165.1 |
|  | S3 | -0.2 | -8.6 | -0.3 | 14.9 | 0 |
|  | S4 | -11.7 | -398.6 | 0 | 18.7 | 0 |
|  | S5 | -21.1 | -1133.0 | -6.6 | 37.7 | 0 |

1. **Subject-specific static non-linearity**

**
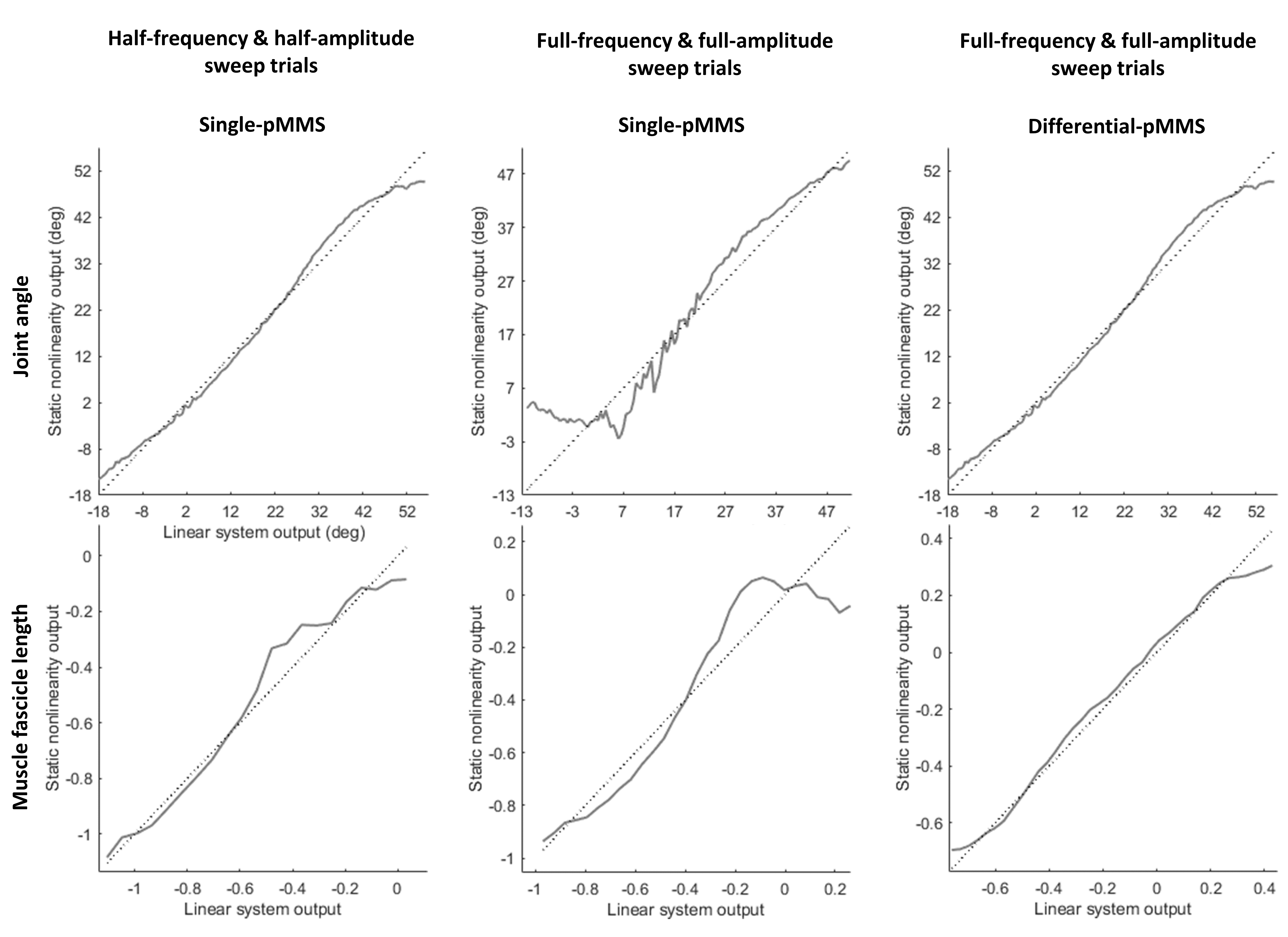
S1 S2**

**
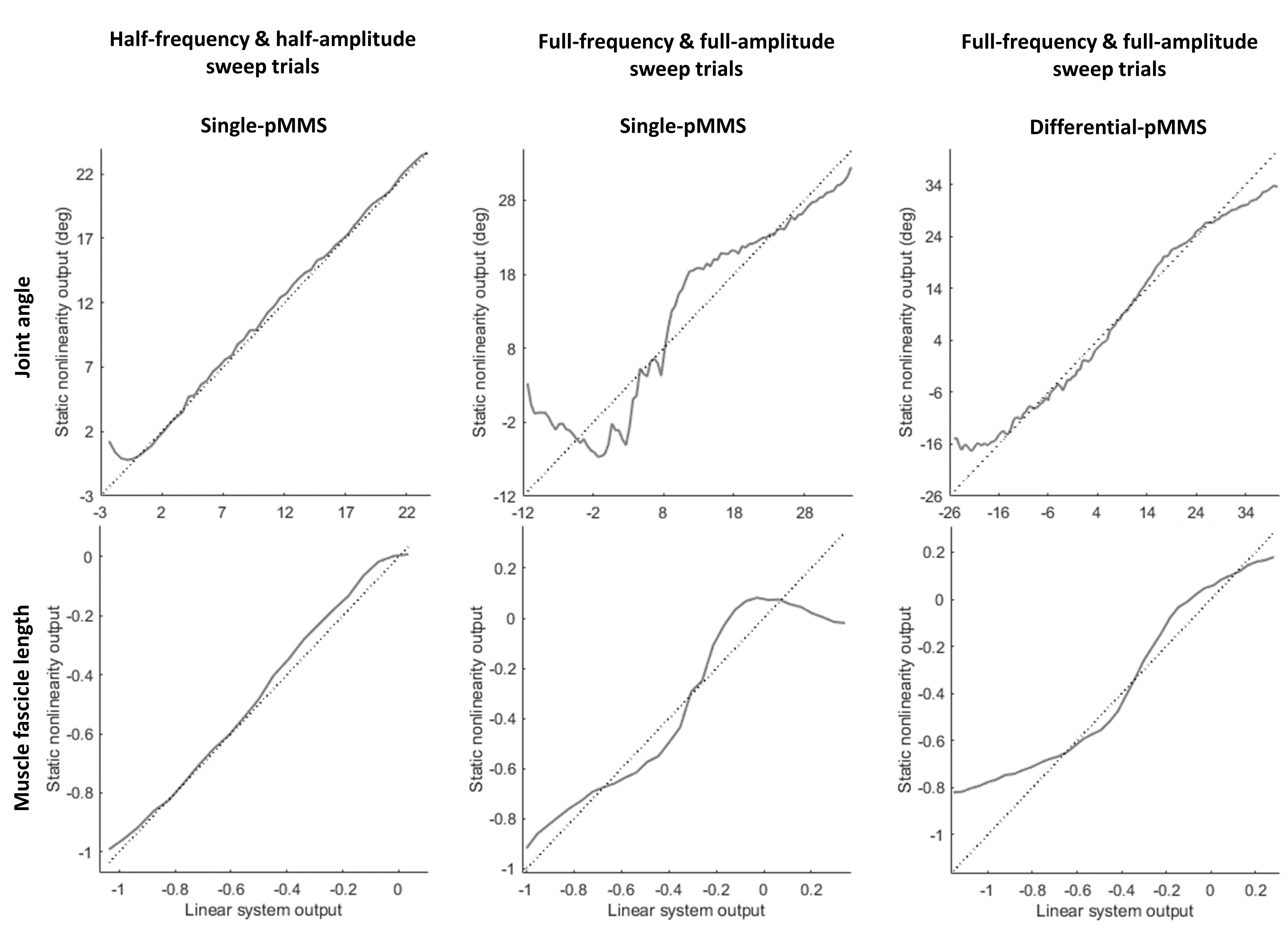
**

**S3 S4**

**
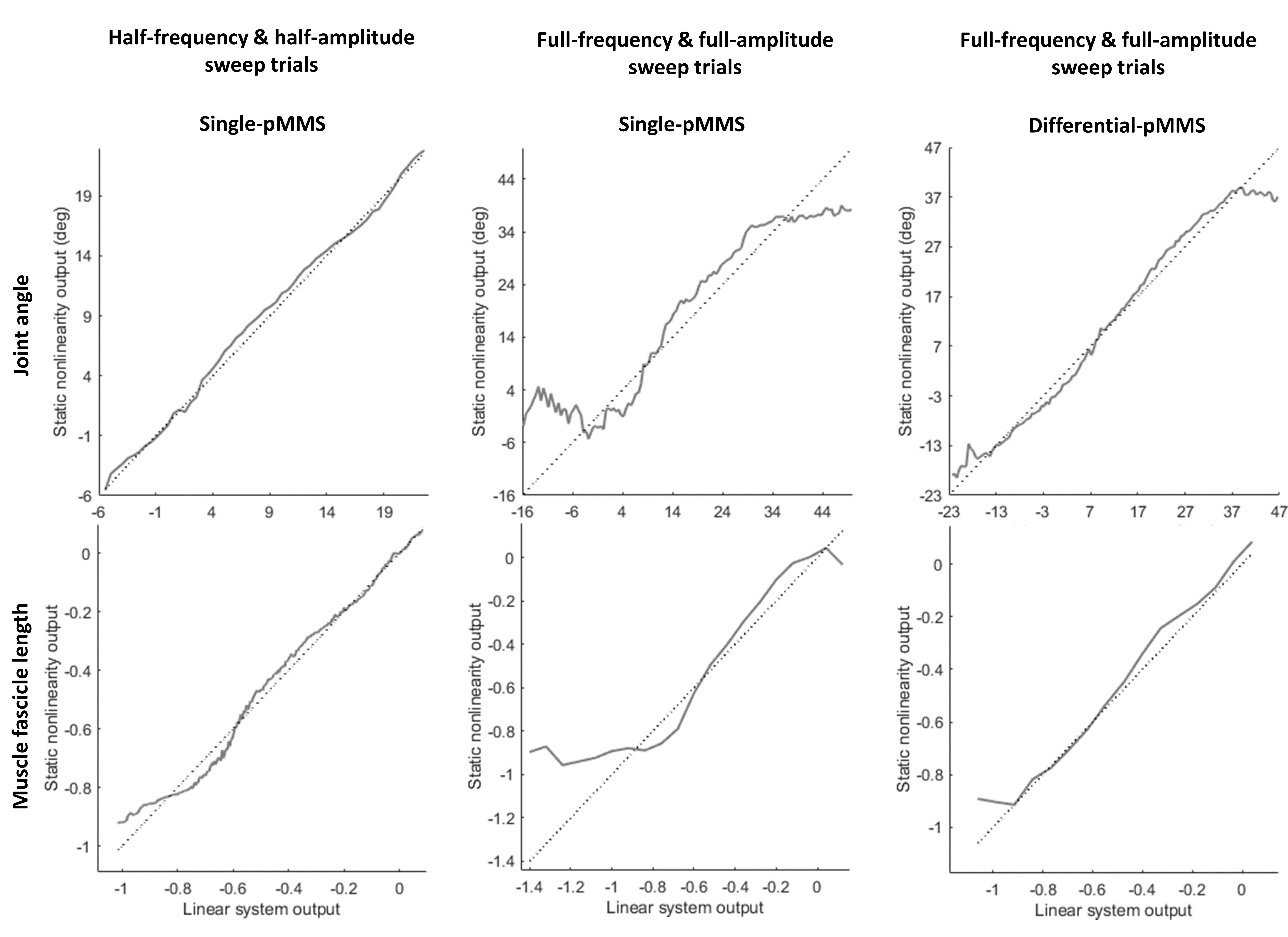

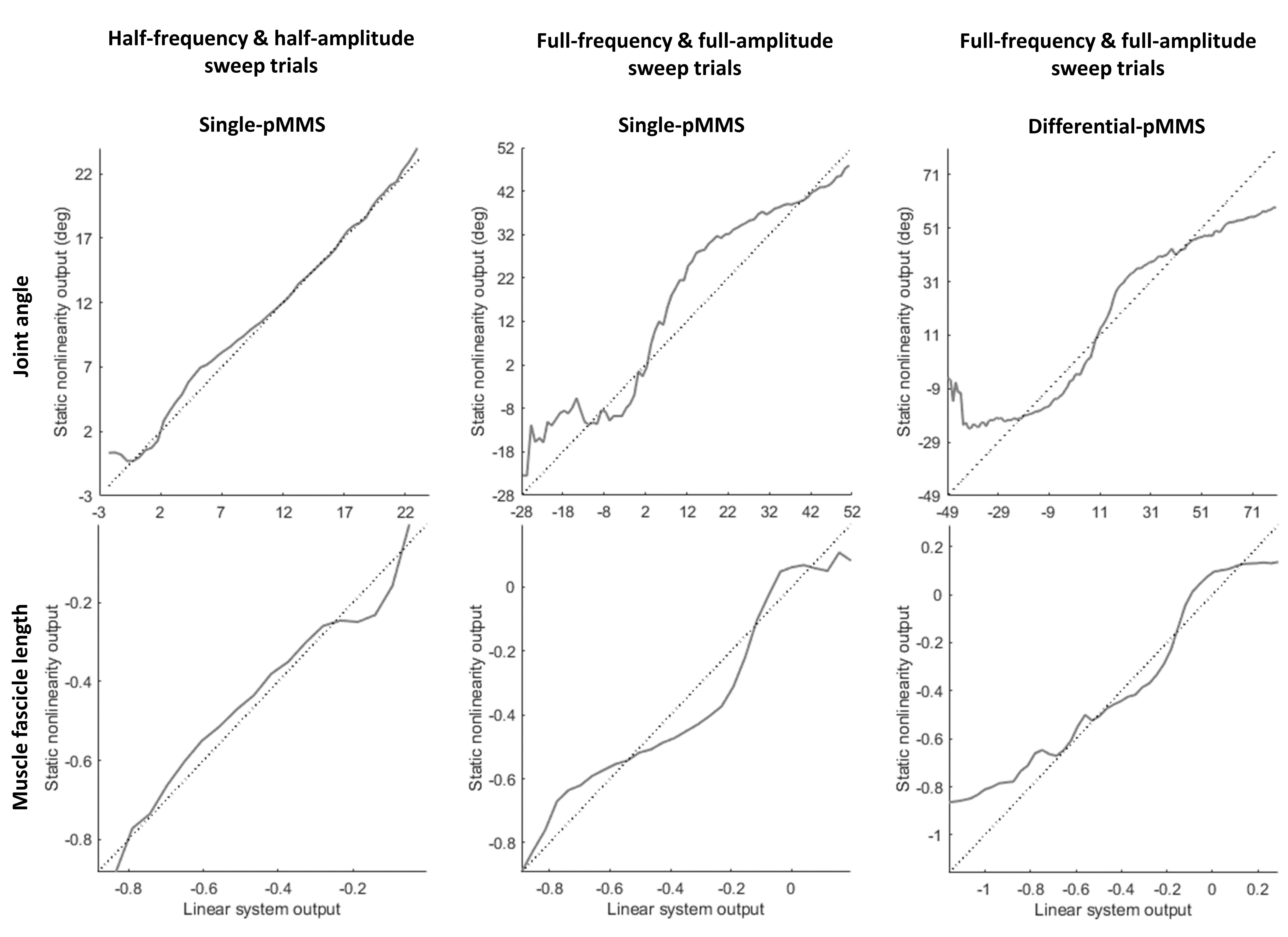
**

**S5**

**
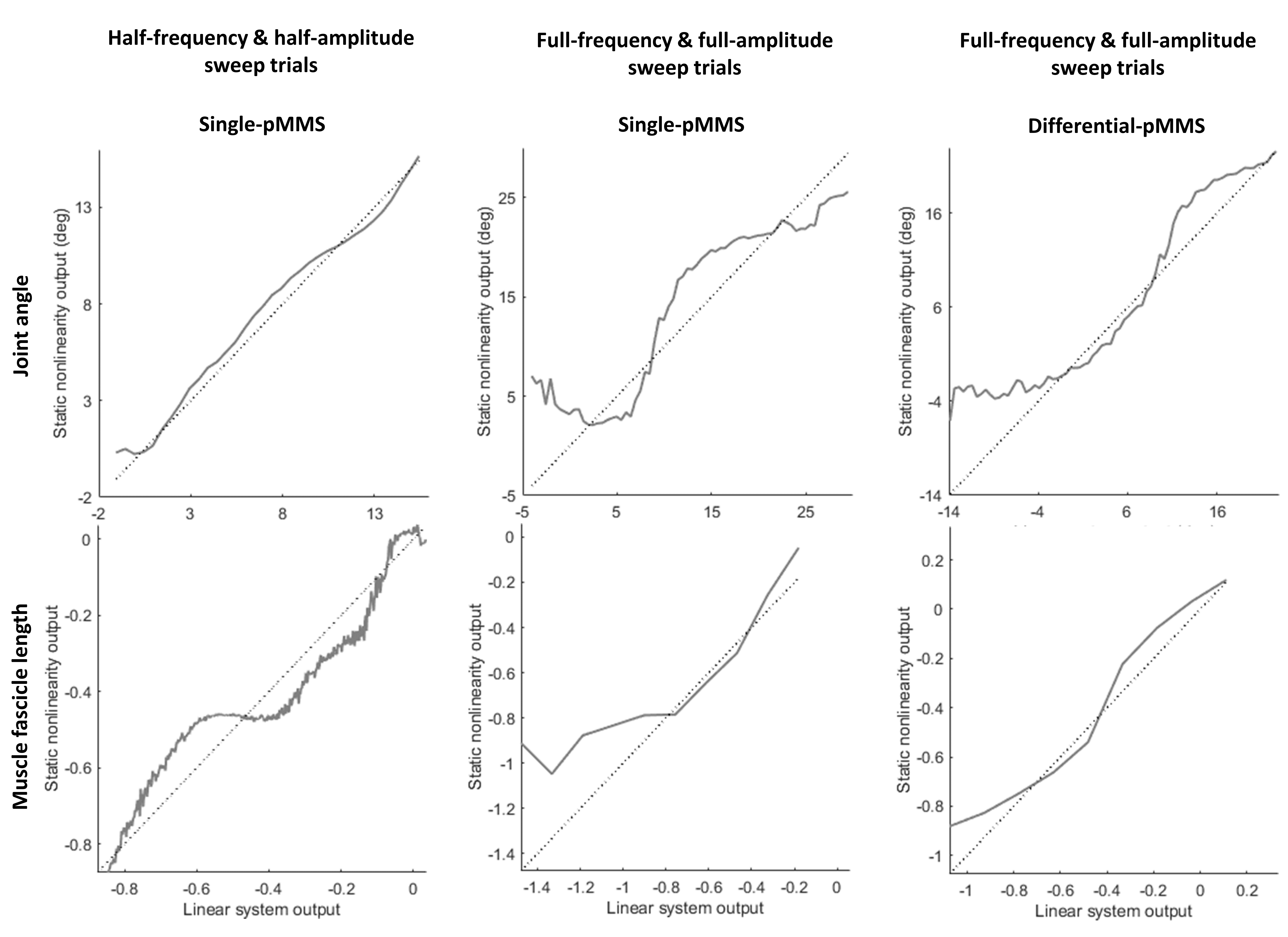
**

1. **Joint angle and muscle fascicle length estimation results (S1 ~ S5)**
2. **Estimation results and linear plots during amplitude and frequency sweep trials**

**
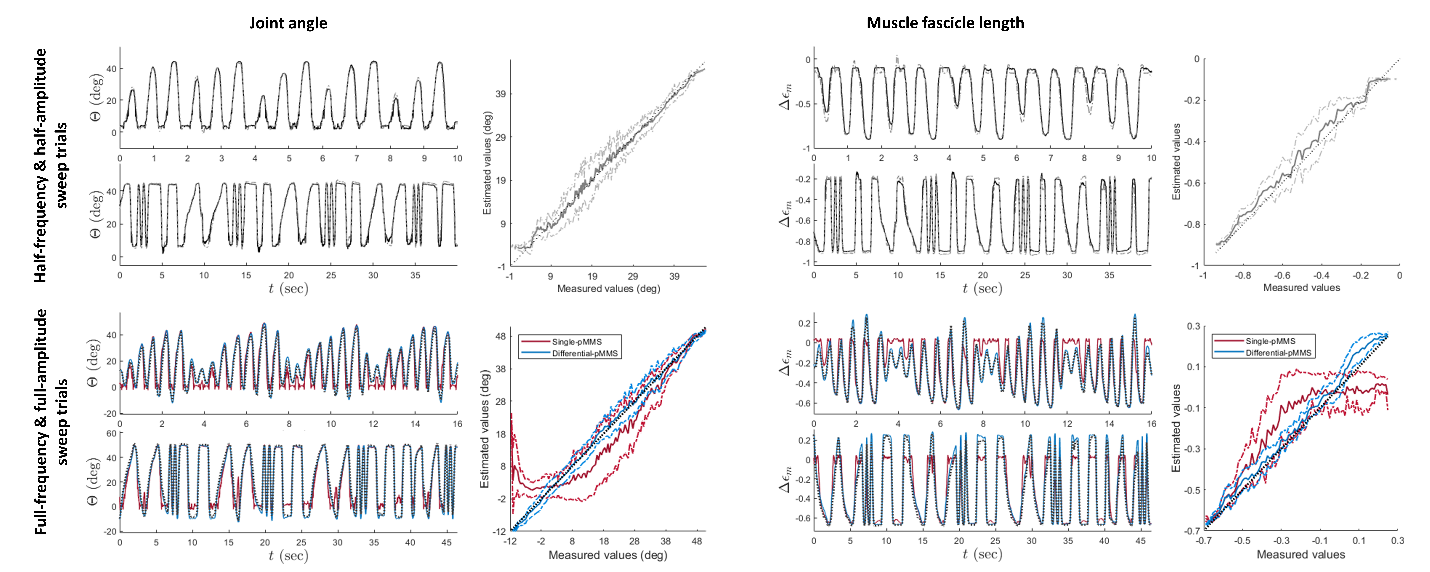
S1**

**
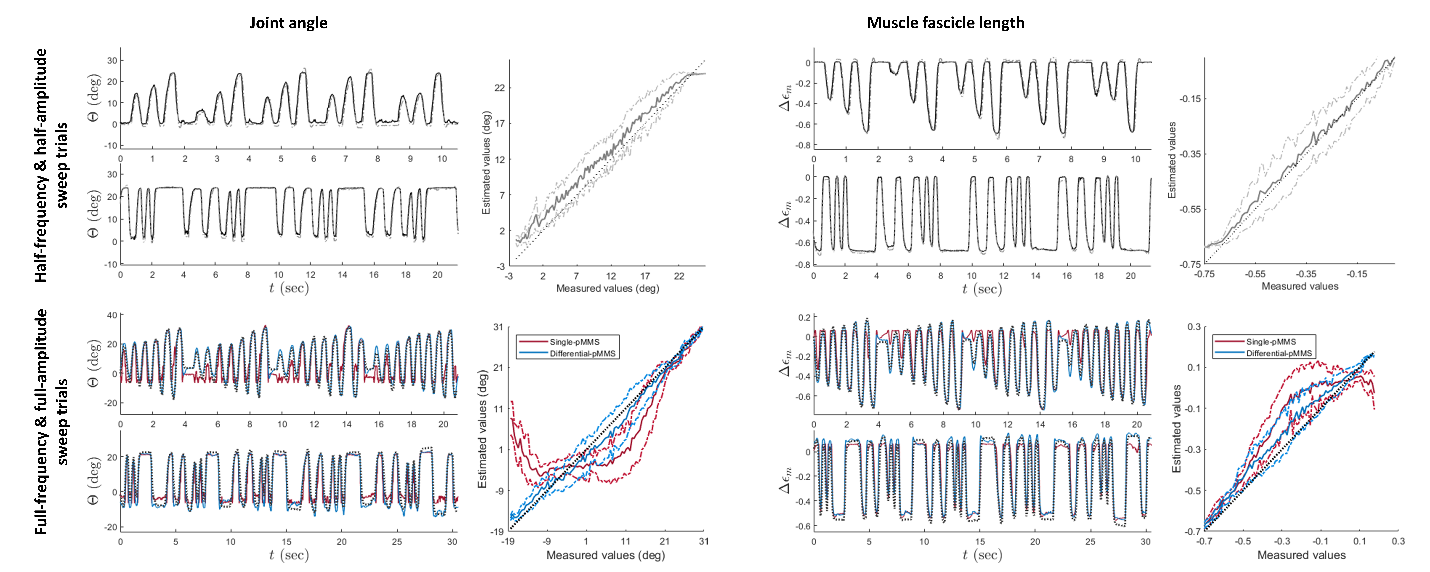
S2**

**
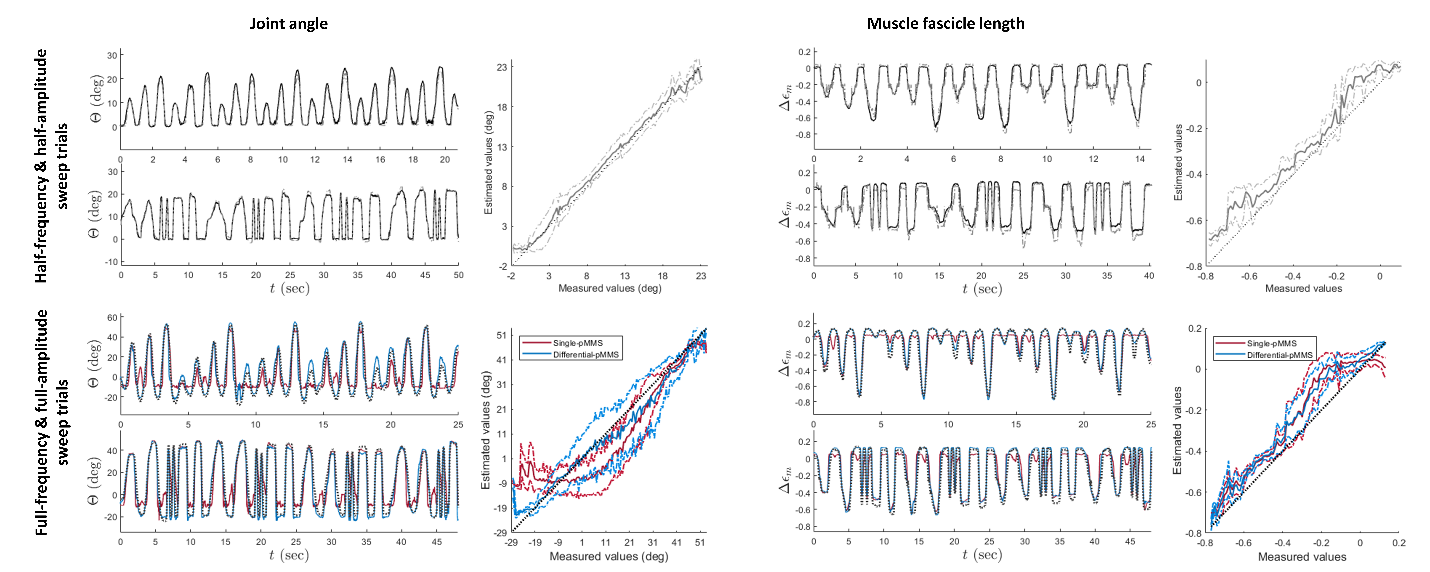
S3**

**
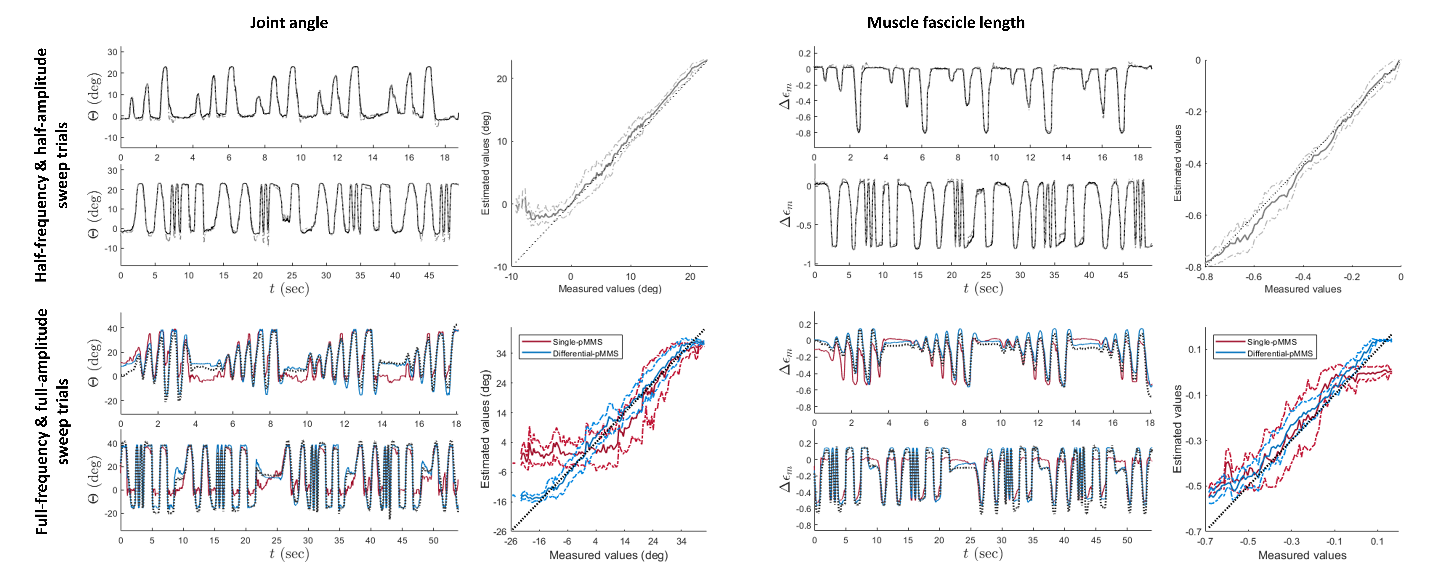
S4**

**
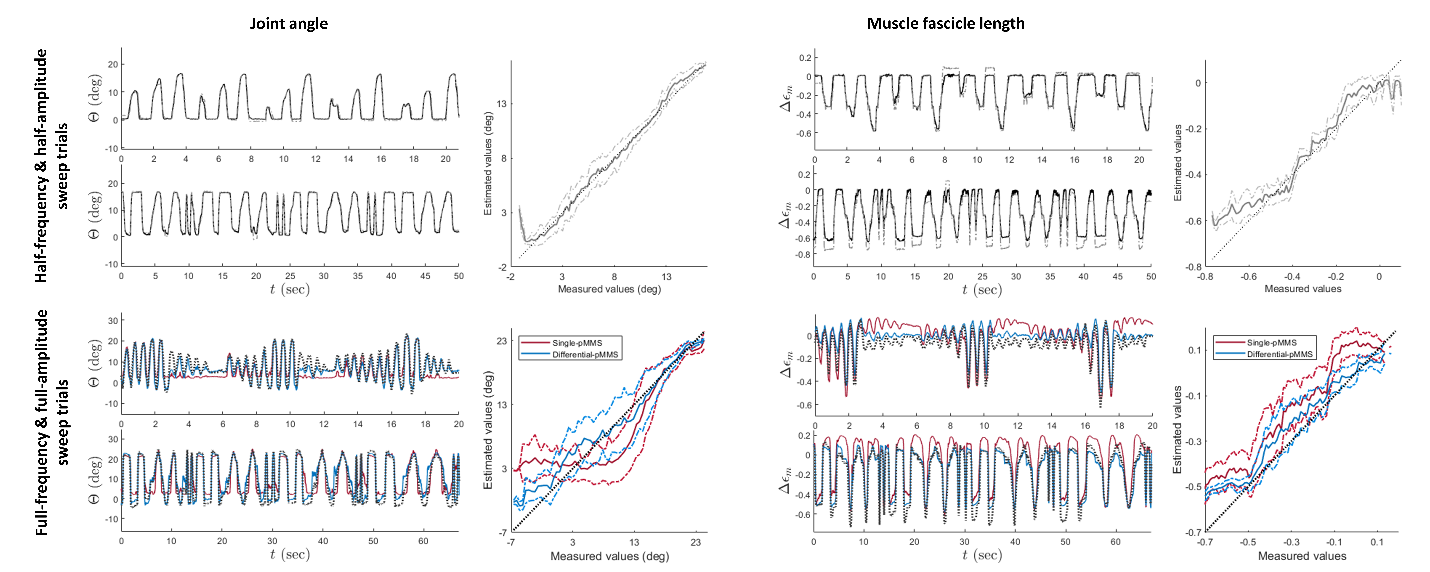
S5**

1. **NRMS errors and R-values of single-pMMS half-amplitude & half-frequency sweep trials (S.H.H.), single-pMMS full-amplitude & full-frequency trials (S.F.F.), and differential-pMMS full-amplitude & full-frequency trials (D.F.F.).**

|  | Subjects | $e_{J}$ | $e_{M}$ | $R_{J}$ | $R_{M}$ |
| --- | --- | --- | --- | --- | --- |
| S.H.H. | S1 | 4.66$\pm$4.63 % | 5.18$\pm$4.83 % | 0.991 | 0.988 |
|  | S2 | 5.69$\pm5$.50 % | 4.65$\pm$4.60 % | 0.988 | 0.998 |
|  | S3 | 4.65$\pm$4.55 % | 7.10$\pm$5.99 % | 0.989 | 0.963 |
|  | S4 | 4.54$\pm$4.23 % | 3.41$\pm$3.27 % | 0.991 | 0.995 |
|  | S5 | 4.50$\pm$4.48 % | 7.94$\pm$6.83 % | 0.992 | 0.968 |
| S.F.F. | S1 | 11.4$\pm11$.4 % | 13.81$\pm13$.73 % | 0.940 | 0.900 |
|  | S2 | 14.0$\pm$13.7 % | 10.48$\pm$9.81 % | 0.862 | 0.937 |
|  | S3 | 13.7$\pm13$.6 % | 10.35$\pm$10.20 % | 0.898 | 0.930 |
|  | S4 | 14.5$\pm$14.3 % | 10.31$\pm$10.20 % | 0.871 | 0.913 |
|  | S5 | 16.3$\pm16$.1 % | 14.37$\pm$8.68 % | 0.852 | 0.933 |
| D.F.F. | S1 | 3.89$\pm3$.84 % | 4.67$\pm$4.08 % | 0.994 | 0.992 |
|  | S2 | 6.45$\pm$6.25 % | 6.66$\pm5$.80 % | 0.973 | 0.979 |
|  | S3 | 9.09$\pm8$.95 % | 7.84$\pm$6.23 % | 0.957 | 0.974 |
|  | S4 | 5.38$\pm5$.32 % | 5.76$\pm$4.38 % | 0.983 | 0.984 |
|  | S5 | 9.97$\pm9$.62 % | 7.10$\pm$6.56 % | 0.950 | 0.965 |
